# Anti‐Neoplastic Effects of Coffee on Gastrointestinal Cancer as Influenced by the Dietary Background: A Cross‐Sectional Study Based on NHANES 2001–2018

**DOI:** 10.1002/cam4.71612

**Published:** 2026-02-06

**Authors:** Huan Zhang, Chenchen Wang, Ju Zhang, Xiaojing Zhu, Jumei Yin, Nuo Yao, Qimeng Pang, Zhihua Liu, Dawei Wu, Zheyi Han, Lei Shang, Yongquan Shi

**Affiliations:** ^1^ Air Force Medical Center Department of Gastroenterology, Air Force Medical University Beijing China; ^2^ State Key Laboratory of Holistic Integrative Management of Gastrointestinal Cancers and National Clinical Research Center for Digestive Diseases Xijing Hospital of Digestive Diseases, Air Force Medical University Xi’ an China; ^3^ Department of Gastroenterology The 961th Hospital of Joint Logistics Support Force of PLA Qiqihar China; ^4^ Department of Critical Care Medicine The 940th Hospital of Joint Logistics Support Force of PLA Lanzhou Gansu China; ^5^ Postgraduate Department Xi’an Medical University Xi’an Shaanxi China; ^6^ Department of Health Statistics School of Preventive Medicine, Air Force Medical University Xi’an China

**Keywords:** coffee, dietary pattern, gastrointestinal cancer, vegetarian diets

## Abstract

**Background:**

Coffee consumption has been strongly associated with gastrointestinal cancers, but the relationship is controversial. This study seeks to assess their correlation under different dietary backgrounds considering the substantial impact of the food matrix on the effects of bioactive compounds in coffee.

**Methods:**

We selected 29,422 adults from 2001 to 2018 National Health and Nutrition Examination Survey (NHANES), categorizing their dietary backgrounds based on food groups and dietary patterns for the study.

**Results:**

The results showed that the incidence of gastrointestinal cancers was 6.25‰, and the incidence was higher in participants consuming coffee (*p* < 0.001). The adjusted ORs (95% CI) for gastrointestinal cancers risk by coffee consumption with specific dietary habits were 0.820 (0.814–0.826) for a low‐salt diet, 0.703 (0.695–0.710) for drinking tea, and 0.581 (0.576–0.586) for high vegetable intake. Factor analyses identified three dietary patterns, and participants who scored higher in the “Western Pattern” and “Balanced Pattern” had a reduced risk of gastrointestinal cancers after consuming coffee, with ORs (95% CIs) of 0.753 (0.746–0.760) and 0.963 (0.954–0.972), respectively. In contrast, coffee consumption linked to higher gastrointestinal cancer risk in participants scoring high on “Vegetarian pattern” with an OR (95% CI) of 1.707 (1.692–1.721).

**Conclusions:**

The anti‐neoplastic effects of coffee on gastrointestinal cancer are related to dietary background. Based on the findings of this study, we recommend that individuals with dietary patterns classified as “Western” or “Balanced” consume coffee, as it may help reduce the risk of gastrointestinal cancer. Conversely, vegetarians may not experience the same benefits from coffee consumption.

**Abbreviations**
CI: confidence interval
DM: diabetes mellitus
IFG: impaired fasting glucose
M: median
MA: Mexican American
NHANES: National health and nutrition examination survey
NHB: non‐hispanic Black
NHW: non‐hispanic White
OH: other hispanic
OR: other race
PIR: poverty income ratio

## 1 Introduction

Coffee is one of the most widely consumed beverages globally, enjoyed by millions of people daily, with its demand continuing to grow [1]. As a result, even minor health impacts of coffee can have significant public health implications due to its extensive consumer base, making it a topic of considerable interest for researchers. Coffee contains a variety of biologically active ingredients including chlorogenic acid, caffeol, and caffeine, which can both interrupt the cell cycle and interfere with DNA repair, as well as exert antioxidant and other protective effects [2].

Gastrointestinal cancer is one of the leading causes of cancer‐related mortality and is more strongly associated with dietary factors than other types of tumors [3]. Coffee possesses theoretical anti‐neoplastic properties, attributed to the anti‐inflammatory, antioxidant, and antiproliferative actions of its phytoconstituents [4]. However, despite decades of clinical research, findings remain inconsistent [2, 5]. In 2016, the International Agency for Research on Cancer (IARC) classified coffee as “unclassifiable as to its carcinogenicity to humans”, citing insufficient evidence to establish a definitive relationship between coffee consumption and tumor development [6]. Consequently, identifying factors that may influence coffee's potential anti‐neoplastic effects has become an increasingly urgent area of investigation.

Gastrointestinal cancers differ from other digestive tumors in that coffee comes into direct contact with the digestive tract and is absorbed through it. Therefore, the expected functioning of coffee is also influenced by the environment in the gastrointestinal tract (physicochemical properties and intestinal flora) as well as by the interaction with other foods and nutrients (e.g., milk, fats, etc.) [7, 8]. Given the critical role of the physicochemical environment and the food matrix in modulating the bioavailability of coffee's active compounds, a pertinent question arises: do different dietary backgrounds limit the “well‐known” anti‐neoplastic effects of coffee?

Research examining the association between coffee consumption and gastrointestinal cancer across varying dietary contexts remains limited. We propose to use the NHANES database to explore the association between coffee consumption and gastrointestinal cancer under different intake situations of foods strongly associated with gastrointestinal cancer (e.g., risk factors: salt [9] and processed meat [10]; protective factors: tea [11], vegetables [12] and citrus fruits [13]). Additionally, recognizing that the human diet functions as an integrated whole, this study further evaluates the association between coffee consumption and gastrointestinal cancer under different dietary patterns to provide a more comprehensive understanding.

## 2 Methods

### 2.1 Data Collection and Research Population

**FIGURE 1:**
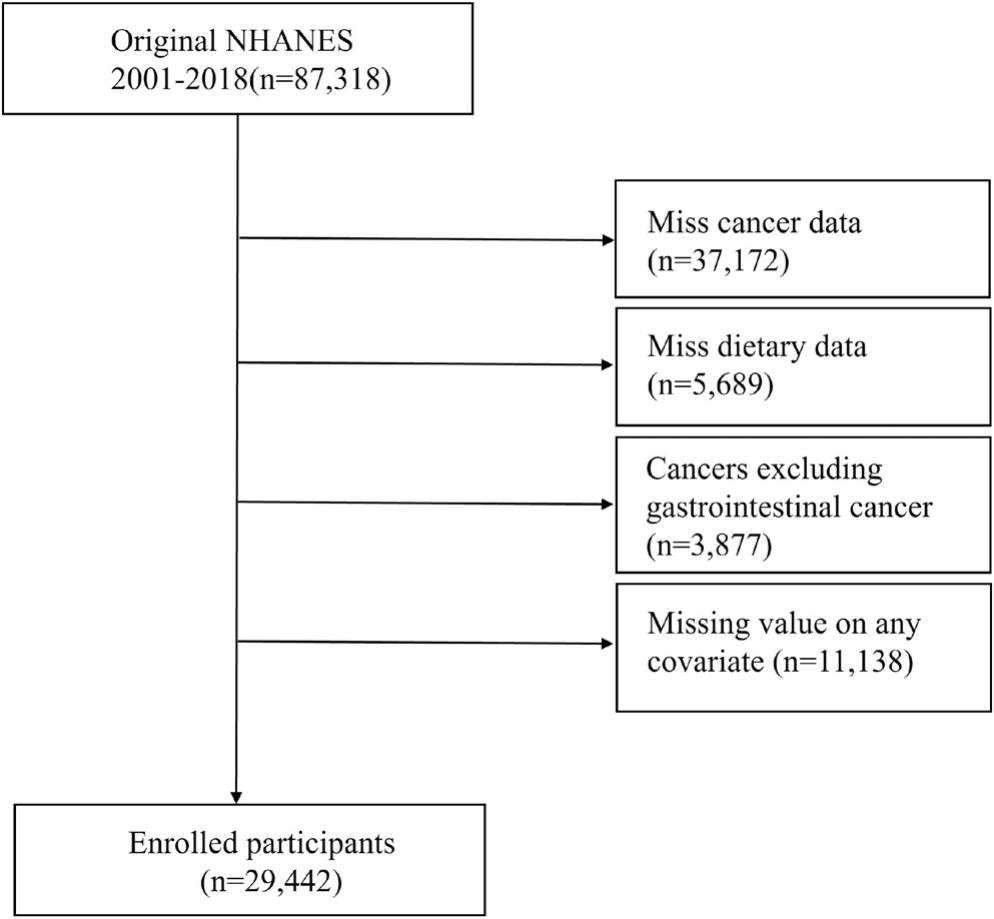
Flow chart for inclusion and exclusion of the study population.

Our samples were obtained from the 2001–2018 National Health and Nutrition Examination Survey (NHANES). NHANES is a continuous survey created and conducted by the Centers for Disease Control and Prevention's (CDC's) National Center for Health Statistics (NCHS). There were a total of 87,318 participants in the nine cycles from 2001 to 2018, excluding participants with missing cancer‐related data (*n* = 37,172), absence of diet and coffee intake (*n* = 5689), exclusion of participants with any cancer other than gastrointestinal cancer (*n* = 3877), and missing values on any covariates of the model (*n* = 11,138), resulting in the inclusion of 29,442 eligible participants, all aged 18 years or older (Figure 1).

### 2.2 Gastrointestinal Cancer

The prevalence of tumors in the participants was assessed by the following questionnaires: “Ever told you had cancer or malignancy?” and “What kind of cancer was it?”. The prevalence of gastrointestinal cancer was obtained by combining the respondents’ answers with codes 16, 17 and 35 representing colorectal, esophageal and gastric cancers, respectively.

### 2.3 Consumption of Coffee

Coffee consumption (in grams) was obtained from the first 24‐h dietary recall interview, and the data included information on all food items consumed by the respondent during the 24‐h period. Respondents’ dietary information was linked to the U.S. Department of Agriculture (USDA) Food and Nutritional Database for Dietary Studies (FNDDS), which identifies coffee beverages with a food code number beginning with 921 [14]. Participants who consumed coffee were categorized into two groups based on median coffee intake, with non‐coffee drinkers as an additional category. Thus all participants were categorized into three groups: (1) no coffee intake, (2) 0.23–503.20 g/day, and (3) > 503.2 g/day.

### 2.4 Dietary Pattern

NHANES dietary data obtained through 24‐h dietary recalls were transformed into 37 food groups by the Food Pattern Equivalency Database (FPED) [15]. Deleting the sum (e.g., Total Vegetables) of each food subgroup (e.g., Dark Green Vegetables and Other Vegetables) resulted in 26 food groups [16]. Dietary patterns were extracted using exploratory factor analysis using the intake equivalents of the 26 food groups described above. Fragmentation plots were examined and only factors with eigenvalues greater than 1 were selected. Varimax rotation is used to help interpret identified patterns, thereby minimizing correlations between factors. Factor loadings were examined to identify the most important contributors in each dietary pattern. Factor scores for each dietary pattern were assigned to each individual based on factor loadings and actual food intake. Derived dietary patterns were named individually based on the food with the highest load (load > 0.25) on that factor.

### 2.5 Covariates

Covariates included household demographic information, lifestyle habits, and self‐reported health status. Household demographic information, including sex, age, race, educational attainment, Poverty income ratio(PIR < 1.3, ≥ 1.3 and < 3.5, ≥ 3.5) [17], and body mass index. Drinking status was categorized into two groups: No (including abstained and never) and Yes (currently drinking). Smoking status was categorized as Never (smoked less than 100 cigarettes in life); Former (smoked more than 100 cigarettes in life and smoke not at all now); Now (smoked more than 100 cigarettes in life and smoke some days or every day).

The three food groups we were interested in FPED served as covariates and were used in subgroup analyses; Total Vegetables were categorized into two categories based on intake equivalents by median: Low and High; Citrus fruits and Cured Meat were categorized into two categories based on whether or not they were consumed: No and Yes. Dietary salt use was derived from the interview question “How often is ordinary salt or seasoned salt added in cooking or preparing foods in your household?” Based on participants’ responses of “never, rarely, occasionally, or very often,” salt use was categorized as Low (including never, rarely, occasionally) and High (very often). The NHANES 24‐h dietary recall also provided grams of each beverage, including tea [18], and participants were categorized into two categories based on whether or not the tea was consumed: no and yes.

Current health status was based on self‐reported outcomes, including diabetes (Yes/Impaired Fasting Glucose (IFG)/Impaired glucose tolerance (IGT) /No), hypertension (Yes/No).

### 2.6 Statistical Analysis

Data were acquired and computed using R 4.2.1 software, and all analyses were performed using SPSS 26.0 software. We followed instructions for selecting appropriate sampling weights and combining weights for nine cycles to account for the complex survey design of the NHANES data. Eligible participants were categorized into two groups based on whether they had gastrointestinal cancer.

All of the quantitative data in the study were non‐normally distributed and were therefore expressed using medians and quartiles [M (Q1, Q3)], and comparisons between groups were made using the Mann–Whitney *U*‐test. Categorical variables were described using *n* (%), and comparisons between groups were made using the chi‐square test (χ2). *p* values < 0.05 were considered statistically significant. Multivariable logistic regression was used to test the association between coffee and its association with gastrointestinal cancers under different food intake scenarios.

Each dietary factor was categorized into two grades based on the median (*P*_50_) of the scores, denoted by M1 and M2 (M1 = < *P*_50_, M2 = ≥ *P*_50_percentile), with higher scores indicating higher intake. The association between coffee consumption and the prevalence of gastrointestinal cancer under different dietary profiles was discussed using the rank of the factor scores as a grouping variable.

## 3 Result

### 3.1 Participants’ Characteristics

A total of 29,442 individuals were included in our study. The general characteristics of all eligible participants, categorized by the presence or absence of gastrointestinal cancer, are presented in Table 1. The median age of the participants was 45.0 (32.0, 57.0) years, and the overall weighted prevalence of gastrointestinal cancer was 6.25‰.

**TABLE 1:** Characteristics of the study sample by gastrointestinal cancer^a^.

*n*	Total	without GI cancer	GI cancer	*p*
	29,442	29,213	229	
**Age(year)** ^b^	45.0 (32.0,57.0)	45.0 (32.0,57.0)	70.0 (57.0,78.0)	< 0.001
**Gender, %**				< 0.001
Female	50.4	50.4	53.2	
Male	49.6	49.6	46.8	
**Race, %**				< 0.001
MA	8.4	8.4	2.1	
NHB	11.3	11.3	10.9	
NHW	68.4	68.3	81.4	
OH	5.1	5.1	2.2	
OR	6.9	6.9	3.5	
**Education level, %**				< 0.001
Less Than 9th Grade	4.9	4.8	10.4	
9–11th Grade	10.4	10.4	9.6	
High School Grad/GED	23.8	23.8	20.0	
Some College or AA degree	31.9	31.9	37.6	
College Graduate or above	29.0	29.1	22.4	
**BMI**(**Kg/m** ^b^)^b^	27.8 (24.1,32.5)	27.8 (24.1,32.5)	28.0 (25.0,33.1)	< 0.001
**PIR, %**				< 0.001
< 1.3	20.9	20.9	23.1	
≥ 1.3, < 3.5	36.0	35.9	43.3	
≥ 3.5	43.1	43.2	33.5	
**Smoke, %**				< 0.001
Never	55.2	55.3	39.2	
Former	23.5	23.4	44.3	
Now	21.3	21.3	16.5	
**Alcohol user, %**				< 0.001
No	24.0	23.9	45.4	
Yes	76.0	76.1	54.6	
**Hypertension, %**				< 0.001
No	64.2	64.4	28.8	
Yes	35.8	35.6	71.2	
**DM, %**				< 0.001
DM	12.6	12.5	29.1	
IFG	4.3	4.3	9.9	
IGT	3.0	3.0	2.5	
No	80.1	80.2	58.5	
**Tea consumption, %**				< 0.001
No	73.4	73.4	69.0	
Yes	26.6	26.6	31.0	
**Citrus consumption, %**				< 0.001
No	69.7	69.7	63.5	
Yes	30.3	30.3	36.5	
**Cured meat consumption, %**				< 0.001
No	52.6	52.6	55.7	
Yes	47.4	47.4	44.3	
**Vegetable consumption, %**				< 0.001
Low	50.0	50.0	50.4	
High	50.0	50.0	49.6	
**Salt consumption, %**				< 0.001
Low	61.8	61.7	67.1	
High	38.2	38.3	32.9	
**Coffee consumption, %**				< 0.001
No	47.3	47.4	29.0	
0.23–503.20 g/day	26.1	26.0	36.4	
> 503.20 g/day	26.6	26.6	34.6	

Abbreviations: DM, diabetes mellitus; GI, gastrointestinal; IFG, impaired fasting glucose; IGT, impaired glucose tolerance; MA, Mexican American; NHB, non‐hispanic Black; NHW, non‐hispanic White; OH, other hispanic; OR, other race; PIR, poverty income ratio.

^a^ All results were survey‐weighted except for counts of categorical variables.

^b^ Median (25%, 75%).

Compared to participants without gastrointestinal cancer, those with gastrointestinal cancer were older, more predominantly female, less educated, had a higher BMI, had a poorer economic status, infrequent smoking and alcohol consumption, and higher prevalence of hypertension and diabetes. In terms of dietary habits, participants with gastrointestinal cancer consumed tea, coffee, and citrus fruits more frequently, and processed meats and vegetables less frequently. They also consumed less salt in their diet.

### 3.2 Consumption of Coffee Is Associated With an Increased Risk of Gastrointestinal Cancer

Table 2 shows the association between coffee consumption and gastrointestinal cancer. Both low and high coffee consumption were positively associated with the overall prevalence of gastrointestinal cancer (*p* < 0.001). After adjusting for covariates, coffee consumption remained a risk factor for gastrointestinal cancer (*p* < 0.001). The prevalence of gastrointestinal cancer in participants who consumed 0.23–503.2 g of coffee per day was 1.26 times higher than in participants who did not consume coffee (95% CI, 1.251–1.265). The prevalence of gastrointestinal cancer in participants who consumed > 503.2 g of coffee per day was 1.20 times higher than in participants who did not consume coffee (95% CI, 1.195–1.209). This result indicates that, when not considering dietary background, coffee consistently represents a risk factor for gastrointestinal cancer.

**TABLE 2:** Associations between consumption of coffee and gastrointestinal cancer^a^.

	Coffee consumption		
	No coffee	0.23–503.20 g/day	> 503.20 g/day
	*n* = 14,306,47.3%	*n* = 8625,26.1%	*n* = 6511,26.6%
*n* [Cancer/without cancer(weighted n for cancer/without cancer)]	66/14,240 (242,127/66,880,811)	91/8534 (303,908/37,082,647)	72/6439 (289,478/37,547,460)
Model1	Ref.	2.282 (2.270,2.295)	2.130 (2.118,2.141)
Model2	Ref.	1.239 (1.232,1.246)	1.177 (1.170,1.184)
Model3	Ref.	1.258 (1.251,1.265)	1.202 (1.195,1.209)

*Note:* Model 1: unadjusted; Model 2: adjusted by age, sex, race, poverty income ratio, education level, alcohol consumption status, smoking status, body mass index, Hypertension, Diabetes; Model 3:Additionally adjusted for salt intake, vegetable intake, citrus intake, cured meat intake and tea consumption.

^a^ All results were survey‐weighted except for sample counts.

### 3.3 Subgroup Analysis

Subgroup analyses are presented in Table 3. In age and sex subgroup analyses, participants older than 60 years and men had a lower prevalence of gastrointestinal cancer at high coffee intake, with ORs of 0.915 (95% CI, 0.909–0.921) and 0.973 (95% CI, 0.964–0.981), respectively. In subgroup analyses based on different dietary habits, different intakes of alcohol, citrus fruits, and processed meat did not alter the original effect of coffee, which remained a risk factor for gastrointestinal cancer at low and high intakes (*p* < 0.001). In contrast, participants who consumed > 503.2 g of coffee per day had significantly lower gastrointestinal risk in the low‐salt and tea consumption groups, with ORs of 0.820 (95% CI, 0.814–0.826) and 0.703 (95% CI, 0.814–0.826), respectively. Coffee was consistently a protective factor against gastrointestinal cancer in participants with higher vegetable intake, with ORs decreasing from 0.838 (95% CI, 0.831–0.845) to 0.581 (95% CI, 0.576–0.586) with increasing coffee intake.

**TABLE 3:** Multivariable‐adjusted odds ratio (95% confidence intervals) of gastrointestinal cancer by consumption of coffee in subgroups^a^.

		Coffee consumption		
Subgroup		No coffee	0.23–503.20 g/day	> 503.20 g/day
**Age**				
*n* [Cancer/without cancer(weighted n for cancer/without cancer)]	< 60	14/11,408 (57,927/57,959,486)	12/5152 (62,663/26,456,510)	19/4032 (99,599/27,082,000)
	≥ 60	52/2832 (184,199/8,921,325)	79/3382 (241,245/10,322,229)	53/2426 (189,879/10,465,460)
OR (95% CI)	< 60	Ref.	2.109 (2.085, 2.134)	2.359 (2.334, 2.386)
	≥ 60	Ref.	1.021 (1.015, 1.028)	0.915 (0.909, 0.921)
**Sex**				
*n* [Cancer/without cancer(weighted n for cancer/without cancer)]	Female	30/7060 (136,675/33,127,370)	45/4675 (154,639/20,696,391)	30/2868 (153,246/17,307,281)
	Male	36/7180 (105,451/33,753,441)	46/3859 (149,269/16,082,348)	42/3572 (136,231/20,240,178)
OR (95% CI)	Female	Ref.	1.120 (1.112, 1.129)	1.466 (1.455, 1.478)
	Male	Ref.	1.442 (1.429, 1.454)	0.973 (0.964, 0.981)
**Salt**				
*n* [Cancer/without cancer(weighted n for cancer/without cancer)]	Low intake	43/8271 (193,351/42,041,803)	66/4685 (219,406/21,884,172)	39/3827 (147,763/23,227,505)
	High intake	23/5969 (48,775/24,839,007)	25/3849 (84,502/14,894,567)	33/2612 (141,715/14,319,955)
OR (95% CI)	Low intake	Ref.	1.152 (1.145, 1.160)	0.820 (0.814, 0.826)
	High intake	Ref.	1.729 (1.709, 1.749)	2.584 (2.555, 2.612)
**Tea**				
*n* [Cancer/without cancer(weighted n for cancer/without cancer)]	No	44/10,177 (143,413/47,203,033)	67/6517 (196,459/27,621,294)	59/5036 (236,568/28,793,084)
	Yes	22/4063 (98,714/19,677,778)	24/2017 (107,449/9,157,445)	13/1403 (52,910/8,754,376)
OR (95% CI)	No	Ref.	1.299 (1.289, 1.308)	1.538 (1.527, 1.549)
	Yes	Ref.	1.322 (1.310, 1.334)	0.703 (0.695, 0.710)
**Alcohol**				
*n* [Cancer/without cancer(weighted n for cancer/without cancer)]	No	38/4461 (112,576/17,963,504)	46/2683 (146,007/8,557,641)	31/1651 (120,891/7,221,652)
	Yes	28/9779 (129,550/48,917,307)	45/5851 (157,901/28,221,098)	41/4788 (168,586/30,325,807)
OR (95% CI)	No	Ref.	1.390 (1.378, 1.401)	1.393 (1.381, 1.406)
	Yes	Ref.	1.130 (1.122, 1.139)	1.075 (1.067, 1.083)
**Vegetable**				
*n* [Cancer/without cancer(weighted n for cancer/without cancer)]	Low intake	36/7789 (92,801/34,886,720)	51/4510 (149,604/18,288,204)	38/3184 (178,876/17,425,298)
	High intake	30/6451 (149,326/31,994,090)	40/4024 (154,304/18,490,535)	34/3255 (110,602/20,122,161)
OR (95% CI)	Low intake	Ref.	2.003 (1.986, 2.021)	2.482 (2.461, 2.503)
	High intake	Ref.	0.838 (0.831, 0.845)	0.581 (0.576, 0.586)
**Citrus**				
*n* [Cancer/without cancer(weighted n for cancer/without cancer)]	No	45/10,491 (154,153/48,564,582)	59/5810 (195,749/24,208,490)	45/4489 (180,788/25,654,514)
	Yes	21/3749 (87,973/18,316,229)	32/2724 (108,159/12,570,250)	27/1950 (108,689/11,892,946)
OR (95% CI)	No	Ref.	1.428 (1.418, 1.438)	1.263 (1.253, 1.272)
	Yes	Ref.	1.015 (1.005, 1.025)	1.168 (1.157, 1.180)
**Cured meat**				
*n* [Cancer/without cancer(weighted n for cancer/without cancer)]	No	32/7785 (135,770/34,796,002)	52/4789 (184,170/20,137,606)	36/3300 (145,190/19,298,808)
	Yes	31/6455 (106,357/32,084,808)	39/3745 (119,738/16,641,133)	36/3139 (144,287/18,248,652)
OR (95% CI)	No	Ref.	1.421 (1.411, 1.432)	1.098 (1.089, 1.107)
	Yes	Ref.	1.088 (1.078, 1.098)	1.318 (1.306, 1.329)

*Note:* Model was adjusted for age, sex, race, poverty income ratio, education level, alcohol consumption status, smoking status, body mass index, Hypertension, Diabetes, salt intake, vegetable intake, citrus intake, cured meat intake and tea consumption.

^a^ All results were survey‐weighted except for sample counts.

### 3.4 Description of Dietary Patterns

Table 4 presents the three dietary patterns extracted by factor analysis following varimax rotation and the main food factor loadings for each pattern. The total variance explained by these three dietary patterns was 22.5%. Based on the contribution to the total variance, the three dietary patterns were: Factor 1 was defined as the “Western pattern” characterized by a dietary intake dominated by solid fats, refined grains, cheese, added sugar, orange vegetables, and processed meats; Factor 2 was defined as the “Vegetarian pattern” characterized by a dietary intake dominated by fruits, other vegetables, orange vegetables, dark green vegetables, whole grains, and yogurt intake; Factor 3 is identified as the “Balance pattern”, which is characterized by dietary intake dominated by intake of vegetable oils, nuts, starchy vegetables, eggs, seafood, and other vegetables.

**TABLE 4:** Factor loadings for food intake patterns in the US National Health and Nutrition Examination Survey (NHANES) 2001–2018.

Food groupings	F1:Western pattern	F2:Vegetarian pattern	F3:Balance pattern
Solid fats	**0.826**	−0.174	0.049
Refined grains	**0.736**	0.009	−0.035
Cheese	**0.678**	0.079	−0.135
Added sugars	**0.483**	−0.224	0.112
Cured meat (frankfurters, sausage, corned beef, cured ham and luncheon meat made from beef, pork, poultry)	**0.299**	−0.132	−0.001
Milk	0.245	0.045	0.033
Other fruits (excluding citrus, melons, and berries)	−0.055	**0.466**	−0.003
Starchy vegetables	0.079	**−0.442**	**0.381**
Red and orange vegetables	**0.386**	**0.432**	−0.015
Other vegetables	0.207	**0.413**	**0.255**
Whole grains	−0.020	**0.394**	0.180
Dark green vegetables	−0.080	**0.384**	0.144
Yogurt	−0.034	**0.368**	−0.087
Red Meat (beef, veal, pork, lamb, game)	0.240	**−0.278**	0.040
Citrus, melons, and berries	−0.039	**0.274**	0.031
Legumes	0.175	0.231	−0.036
Soy Products	−0.036	0.221	−0.018
Seafood high in n‐3 fatty Acids	−0.059	0.215	0.083
Fruit juice	0.039	0.143	0.103
Oils	**0.277**	0.101	**0.792**
Nuts and seeds	−0.042	0.172	**0.629**
Eggs	0.215	−0.083	**0.276**
Seafood Low in n‐3 fatty acids	−0.016	0.145	**0.274**
Poultry (chicken, turkey, other fowl)	−0.050	0.037	**0.251**
Alcoholic drinks	0.053	−0.138	0.241
Organ Meat (from beef, veal, pork, lamb, game, poultry)	0.014	−0.004	−0.067
Eigenvalue	2.604	1.855	1.396
Variance explained (%)	10.014	7.136	5.370
Cumulative variance explained (%)	10.014	17.150	22.520

*Note:* Factor loadings > 0.25 or < −0.25 are in bold type.

### 3.5 Consumption of Coffee Is Associated With a Reduced Risk of Gastrointestinal Cancer According to Dietary Patterns

The association between coffee consumption and gastrointestinal cancer based on dietary patterns is shown in Table 5. Both low and high coffee consumption showed a protective effect at higher Western pattern scores, with ORs of 0.772 (95% CI, 0.765–0.780) and 0.963 (95% CI, 0.954–0.972), respectively. At low “Vegetarian pattern” scores, coffee eventually became a protective factor against gastrointestinal cancer as intake increased, with an OR of 0.900 (95% CI, 0.892–0.907). At high “Balance pattern” scores, high coffee intake significantly reduced the risk of gastrointestinal cancer, with an OR of 0.753 (95% CI, 0.746–0.760).

**TABLE 5:** The association between drinking coffee and gastrointestinal cancer by dietary pattern score median^a^.

Dietary pattern		Coffee consumption			*p*
		No coffee	0.23–503.20 g/day	> 503.20 g/day	
**Western pattern**					
*n* [Cancer/without cancer(weighted n for cancer/without cancer)]	**M1**	45/7458 (135,675/32,392,837)	68/5045 (225,355/20,708,018)	44/3115 (173,502/17,396,788)	
	**M2**	21/6782 (106,451/34,487,974)	23/3489 (78,553/16,070,721)	23/3324 (115,976/20,150,671)	
OR (95% CI)	**M1**	Ref.	1.644 (1.632,1.656)	1.398 (1.387,1.408)	< 0.001
	**M2**	Ref.	0.772 (0.765,0.780)	0.963 (0.954,0.972)	< 0.001
**Vegetarian pattern**					
*n* [Cancer/without cancer(weighted n for cancer/without cancer)]	**M1**	41/7709 (145,361/35,711,447)	53/3938 (154,709/16,451,453)	35/3234 (132,386/18,427,151)	
	**M2**	25/6531 (96,765/31,169,364)	38/4596 (149,199/20,327,286)	37/3205 (157,092/19,120,309)	
OR (95% CI)	**M1**	Ref.	1.079 (1.071,1.087)	0.900 (0.892,0.907)	< 0.001
	**M2**	Ref.	1.497 (1.484,1.510)	1.707 (1.692,1.721)	< 0.001
**Balance pattern**					
*n* [Cancer/without cancer(weighted n for cancer/without cancer)]	**M1**	32/7467 (136,506/34,344,194)	53/4736 (149,081/18,635,858)	45/3179 (194,405/17,562,087)	
	**M2**	34/6773 (105,621/32,536,617)	38/3798 (154,827/18,142,881)	27/3260 (95,073/19,985,373)	
OR (95% CI)	**M1**	Ref.	1.032 (1.024,1.040)	1.657 (1.644,1.669)	< 0.001
	**M2**	Ref.	1.589 (1.576,1.603)	0.753 (0.746,0.760)	< 0.001

*Note:* Model was adjusted for age, sex, race, poverty income ratio, education level, alcohol consumption status, smoking status, body mass index, hypertension, diabetes, salt intake and tea consumption.

Abbreviation: M, median.

^a^ All results were survey‐weighted except for sample counts.

## 4 Discussion

This study explored the association between coffee consumption and gastrointestinal cancer risk in various dietary contexts using a representative sample of U.S. adults. We found that coffee consumption was positively associated with the prevalence of gastrointestinal cancer overall. Subgroup analyses revealed that coffee consumption significantly reduced the risk of gastrointestinal cancer in participants with low salt intake and higher consumption of tea and vegetables. Taking a more holistic view from the perspective of dietary structure, in short, the “Western pattern” and “Balance pattern” contributed to the anti‐neoplastic effect of coffee on gastrointestinal cancers, while the “Vegetarian pattern” limited this effect.

Just as the effects of coffee are often dependent on age, gender, source, and intake volume, we found differences in its age and gender subgroups [19]. Coffee metabolism is more complex in women due to estrogenic influence [20]. Coffee can inhibit cancer through the anti‐estrogenic pathway, but at the same time, some of the active components of coffee also have estrogenic activity, binding to estrogen receptors to activate signaling pathways [19, 21]. Most studies have focused on metabolic diseases and female‐specific cancers (e.g., ovarian and breast cancers), with fewer examining its effects on gastrointestinal cancers. Further research is needed to better understand the role of coffee in these cancers.

We grouped participants by diet and found that a low‐salt diet, consumption of tea and more vegetables may help coffee exert its anti‐neoplastic effects. High‐salt diets can be involved in gastrointestinal diseases by disrupting intestinal immune homeostasis and disturbing the intestinal microbiota, and it remains unknown whether the remodeling of the gastrointestinal environment affects coffee absorption and metabolism [22]. Phenolic compounds in various plant foods differ dramatically in chemical structure, but the cells are all homogeneously involved in antioxidant, detoxification, and repair [23]. Other phenolic or anti‐neoplastic food components (e.g., cinnamon, curcumin, and α‐tocopherol) act synergistically to enhance the antioxidant [24] and anti‐genotoxic [25] capacity of coffee. The indigestible fibers in the plant also act as carriers to help the polyphenols deliver their intended benefits [26]. However, Kolb et al. suggested that the antioxidant and anti‐inflammatory capacity of coffee alone is limited, and thus further studies are needed to explore their interactions [23]. Additionally, the small number of cases in this study limits our ability to make more nuanced classifications, and our results warrant validation through large‐sample prospective studies.

Interestingly, we found that coffee consumption was consistently protective against gastrointestinal cancer in participants with diets that lean toward the “Western pattern.” This pattern, typically linked to an unhealthy diet, is known to create a pro‐inflammatory, oxidative stress environment in the gut [27]. Polyphenols, compounds found in coffee, can act both as antioxidants and pro‐oxidants, and a certain level of oxidative stress may enable these polyphenols to activate the body's antioxidant systems and enhance immune responses [28]. In addition, dietary lipids alter the physical properties of cell membranes (e.g., permeability) and enhance the absorption of certain flavonoids, partially explaining the antioxidant and antimicrobial properties of flavonoids [26]. Isoflavonoids are even more bioavailable in fat‐ and protein‐rich foods than in the absence of food [29]. From a food matrix perspective, combining coffee extract with high‐temperature foods may enhance lipid stability and reduce the production of carcinogenic compounds [30]. Previous studies, such as Kanner et al.'s work on food interactions, have shown that antioxidant‐rich foods (like coffee and red wine) combined with meats can alleviate postprandial oxidative stress [31]. However, it was also noted by the authors that a healthy diet (Mediterranean diet) produces benefits from consuming large amounts of antioxidants, and that food interactions in the stomach are not related to the bioavailability of polyphenols. This indicates that the difference in the anti‐tumor effects of coffee in the “Balance pattern” may come from the small intestine and the colon.

In contrast, coffee did not show significant anti‐neoplastic effects in participants adhering to a “Vegetarian pattern”. This dietary pattern, rich in plant polyphenols, might limit coffee's impact. Studies suggest that high doses of phenolic compounds can cause cytotoxicity, which could undermine the potential health benefits of coffee [23]. Our conclusions in the Vegetarian pattern seem to be inconsistent with the results of the subgroup analyses of vegetables in the single food group, but it is necessary to point out that the most dominant component in the “Vegetarian pattern” is fruit. Research has shown that polyphenols can form polyphenol‐pectin complexes with pectin in fruits and juices, which have no ability to scavenge superoxide and have reduced hydroxyl radical scavenging activity [32]. This implies that the function of the active substances in coffee may be influenced by the food matrix of the plant‐based diet. Furthermore, some people are accustomed to adding chili peppers to their coffee, and the anti‐free radical effects of the two appear synergistic, but are transformed into antagonistic effects after in vitro digestion [24]. This underscores the importance of considering not only the direct interactions between foods but also the digestive action in the gastrointestinal tract.

In summary, this study examined the relationship between coffee consumption and gastrointestinal cancer risk across different dietary patterns among U.S. adults. Several strengths of our study include the use of a representative sample from NHANES and its rigorous quality control standards. Our subgroup analyses took into account both individual food group intakes and overall dietary patterns, offering a more comprehensive view of dietary impacts. At the same time our study has some limitations. First, our study was cross‐sectional and could not assess the causal relationship between coffee consumption and gastrointestinal cancer. Secondly, the 24‐h dietary recall data may not accurately capture long‐term dietary habits. While established eating patterns can persist for years, participants’ dietary habits may change in response to a cancer diagnosis, potentially introducing bias into the study. Similarly, the assessment of cancer was based on self‐report and was not verified with cancer registries or medical records, which may have led to potential misclassification. Third, the factor analysis was a data‐driven exploratory analysis, which may have some limitations when generalizing to other populations.

## 5 Conclusion

In conclusion, our study found that the anti‐neoplastic effect of coffee on gastrointestinal cancer is influenced by the dietary background. Based on the findings of this study, we recommend that individuals with dietary patterns classified as “Western” or “Balanced” consume coffee, as it may help reduce the risk of gastrointestinal cancer. Conversely, vegetarians may not experience the same benefits from coffee consumption. Future studies should pay more attention to the interaction between coffee and food matrix.

## Author Contributions

**Huan Zhang:** conceptualization (equal), formal analysis (equal), writing – original draft (equal). **Chenchen Wang:** conceptualization (equal), writing – original draft (equal). **Ju Zhang:** data curation (equal). **Xiaojing Zhu:** formal analysis (equal). **Jumei Yin:** data curation (equal). **Nuo Yao:** formal analysis (equal). **Qimeng Pang:** data curation (equal). **Zhihua Liu:** formal analysis (equal). **Dawei Wu:** writing – review and editing (equal). **Zheyi Han:** conceptualization (equal), writing – review and editing (equal). **Lei Shang:** conceptualization (equal), formal analysis (equal), writing – original draft (equal), writing – review and editing (equal). **Yongquan Shi:** conceptualization (equal), funding acquisition (equal), writing – review and editing (equal).

## Funding

This work was supported by Shaanxi Key Research and Development Project, 2023‐ZDLSF‐35. Booster Plans of Xijing Hospital, XJZT21L07. Shaanxi Health Scientific Research Innovation Team Project, 2024TD‐06. National Natural Science Foundation of China, 82170560, 82200567, 82330100.

## Ethics Statement

As the data used in this study were obtained from publicly available NHANES data, all data‐related research had previously received approval from their respective ethical review committees and had obtained written informed consent from the participants. Consequently, this study does not require additional ethical approval.

## Consent

We used anonymous de‐identified data that are publicly available from the NHANES. The NHANES obtained informed consent from all participants. Information related to this process can be found at https://wwwn.cdc.gov/nchs/nhanes/. The NHANES obtained informed consent from all participants.

## Conflicts of Interest

The authors declare no conflicts of interest.

## Data Availability

All data of the study are presented in the text or Supporting Information. The datasets analyzed during the current study were publicly available from the NHANES. Data from the NHANES can be found at https://www.cdc.gov/nchs/nhanes/index.htm. The FPED is available at https://www.ars.usda.gov/northeast‐area/beltsville‐md‐bhnrc/beltsville‐human‐nutrition‐research‐center/food‐surveys‐research‐group/docs/fped‐overview/.
